# Tailoring Nutritional Advice for Mexicans Based on Prevalence Profiles of Diet-Related Adaptive Gene Polymorphisms

**DOI:** 10.3390/jpm7040016

**Published:** 2017-11-10

**Authors:** Claudia Ojeda-Granados, Arturo Panduro, Karina Gonzalez-Aldaco, Maricruz Sepulveda-Villegas, Ingrid Rivera-Iñiguez, Sonia Roman

**Affiliations:** 1 Department of Molecular Biology in Medicine, Civil Hospital of Guadalajara “Fray Antonio Alcalde”, Hospital #278, Col. El Retiro, Guadalajara 44280, Jalisco, Mexico; claudiaojedagranados@hotmail.com (C.O.-G.); apanduro@prodigy.net.mx (A.P.); karinaldaco@hotmail.com (K.G.-A.); m_sep03@hotmail.com (M.S.-V.); ingrid_rivei@hotmail.com (I.R.-I.); 2 Health Sciences Center, University of Guadalajara, Guadalajara 44340, Jalisco, Mexico

**Keywords:** nutrigenetics, pre-Hispanic diet, *MTHFR* C677T, *ABCA1* R230C, *APOE* alleles, lactase persistence, *AMY1*, biodiversity, food culture, chronic diseases

## Abstract

Diet-related adaptive gene (DRAG) polymorphisms identified in specific populations are associated with chronic disorders in carriers of the adaptive alleles due to changes in dietary and lifestyle patterns in recent times. Mexico’s population is comprised of Amerindians (AM) and Mestizos who have variable AM, European (EUR) and African genetic ancestry and an increased risk of nutrition-related chronic diseases. Nutritional advice based on the Mexican genome and the traditional food culture is needed to develop preventive and therapeutic strategies. Therefore, we aimed to provide a prevalence profile of several DRAG polymorphisms in the Mexican population, including Central West (CW) Mexico subpopulations. Geographic heat maps were built using ArcGIS10 (Esri, Redlands, CA, USA) software, based on the published data of the *MTHFR* C677T (rs1801133), *ABCA1* Arg230Cys (rs9282541), *APOE* T388C (rs429358)/C526T (rs7412), *LCT* C-13910T (rs4988235) polymorphisms and *AMY1* copy number variation (CNV). Also, new data obtained by allelic discrimination-real-time polymerase chain reaction (RT-PCR) assays for the *MTHFR*, *ABCA1*, and *APOE* polymorphisms as well as the *AMY1* CNV in the CW Mexico subpopulations with different proportions of AM and EUR ancestry were included. In the CW region, the highest frequency of the *MTHFR* 677T, *ABCA1* 230C and *APOE* ε4 adaptive alleles was observed in the AM groups, followed by Mestizos with intermediate AM ancestry. The *LCT*-13910T allele frequency was highest in Mestizos-EUR but extremely low in AM, while the *AMY1* diploid copy number was 6.82 ± 3.3 copies. Overall, the heat maps showed a heterogeneous distribution of the DRAG polymorphisms, in which the AM groups revealed the highest frequencies of the adaptive alleles followed by Mestizos. Given these genetic differences, genome-based nutritional advice should be tailored in a regionalized and individualized manner according to the available foods and Mexican traditional food culture that may lead to a healthier dietary pattern.

## 1. Introduction

Traditional diets and lifestyles in several societies around the world have endured a dramatic nutrition transition in recent years. This nutrition transition has contributed to the present-day obesity pandemic and rising incidence of nutrition-related non-communicable chronic diseases (NR-NCD) [1,2]. The mechanisms underlying these diseases are part of an unfavorable balance between the human genome and the current environment, due to diet-related genetic adaptations to past nutritional regimes [2,3]. Genes evolve to allow humans to adapt to a vast diversity of natural environments (climates, food sources, pathogens) [4]. However, diet and lifestyle have been among the most important natural and cultural selection pressures influencing genetic variability and differentiation among stable human populations. Thereby, gene-culture co-evolution can affect the rate at which allele frequencies change in a population in response to a particular environment as influenced by cultural practices [5].

Progress in genetics and molecular biology methodologies, together with anthropological and archaeological studies have shown evidence of these gene-culture co-evolutionary processes [6,7]. For example, signatures of genetic adaptation were identified in genes involved in food-related metabolic pathways as a response to human cultural practices [8,9]. One of the best-known cases of diet-related adaptive gene (DRAG) polymorphisms are the genetic variants in the promoter region of the *LCT* gene, which encodes for the lactase enzyme. These variants are associated with adult lactase persistence in some populations of Northern Europe, Africa, and the Middle East, which have had a traditional culture of pastoralism and milk consumption [10,11]. Another example is the variation in *AMY1* gene copy number that encodes salivary amylase for starch digestion. A higher average *AMY1* copy number, positively correlated with a higher expression of the enzyme, has been observed in agricultural societies such as European-Americans and Japanese that historically consume starch-rich diets [12].

In Native American populations, gene-culture co-evolutionary processes may also have occurred [13]. Notably, the pre-Hispanic civilizations of Mexico are highly recognized for their food culture, healthy lifestyle and nutrition [14]. The emergence of plant domestication and sophisticated agricultural systems in Mesoamerica, which took place at least 5000 years ago, marked the starting point for the establishment of many ancient civilizations and a new food culture. The Mesoamericans consumed a pre-Hispanic diet comprised of three essential components: maize, beans, and squash, enriched with diverse chili plants, a wide variety of endemic leafy green vegetables denoted as “quelites”, tomatoes, amaranth and chia seeds, algae, insects as well as other vegetables with regional and seasonal variations. Therefore, it is not surprising that diet-related genetic adaptations might have occurred [15,16,17]. Furthermore, genes such as *MTHFR*, *ABCA1*, and *APOE* show a high prevalence of adaptive alleles, of which some are exclusive to Amerindians, and are most likely related to a Mesoamerican co-evolutionary process [18,19,20]. About 500 years ago, the Mexican Mestizo population originated as an admixture between Spanish colonizers, Amerindians (AM) and African slaves. The Mestizo population comprise a variable proportion of AM, European (EUR) and African ancestry depending on the geographic region [21]. For instance, Central West (CW) Mexico harbors a heterogeneous population, with ethnic groups having a high amount of AM ancestry and Mestizos having an intermediate AM ancestry or predominantly EUR ancestry (Mestizos-EUR) [22,23,24]. This recent genetic admixture explains the presence in Mexicans of DRAG polymorphisms identified in other populations worldwide. Such is the case, for example, of the *LCT* C-13910T variant related to lactase persistence in EUR, which has been found in Mexican subpopulations with high EUR ancestry but is absent in AM subpopulations [24].

Given the role that diet has played in human genetic adaptability, the study of DRAG polymorphisms could provide the information needed to perform associational and interventional studies to tailor nutritional guidelines to these specific populations. A preliminary approach would be to collate the prevalence profile of DRAG polymorphisms and analyze any regional differences that might occur, which may serve to help provide regional and genome-based nutritional advice to the admixed population of Mexico.

Therefore, this study aimed to provide prevalence profiles of several DRAG polymorphisms in the Mexican population, including CW Mexico subpopulations with different proportions of AM and EUR ancestry.

## 2. Materials and Methods 

### 2.1. Study Population

Two complementary strategies were used to conduct this study. First, a review of the literature was carried out by using electronic databases (PubMed and Google Scholar) to find studies reporting data on the frequency of the DRAG polymorphisms, depicted in Table 1, in healthy AM (ethnic) groups and Mestizo populations of Mexico. If more than one study was found reporting frequencies for the same patient cohort or population by the same authors or research group, then this data was considered only once for that population.

To assess the frequency of DRAG polymorphisms in the CW Mexico region, genotyping of rs1801133, rs9282541, rs429358, and rs7412 and determination of *AMY1* copy number were performed in different subpopulations with DNA samples of unrelated individuals obtained in previous studies by our research group [23,24]. The subpopulations were the ethnic groups Huichol (*n* = 99) and Nahuas (*n* = 84); Mestizos from Tepic in Nayarit State (*n* = 184) and Guadalajara in Jalisco State (*n* = 768); and Mestizos-EUR from the towns Cuquio (*n* = 131), Villa Purificación (*n* = 32) and San Miguel el Alto (*n* = 33) in Jalisco. In previous studies, we showed that these subpopulations have a predominant AM ancestry (ethnic groups), intermediate AM ancestry (Mestizos), and a substantial EUR ancestry (Mestizos-EUR) [23,24].

### 2.2. Genotyping

Genomic DNA was extracted from peripheral blood leukocytes by a modified salting-out procedure and stored at a concentration of 20 ng/μL at −70 °C [25]. Predesigned TaqMan Single Nucleotide Polymorphism (SNP) Genotyping Assays (Applied Biosystems, Life Technologies, Camarillo, CA, USA) were used for the determination of *MTHFR* C677T (C__1202883_20), *ABCA1* Arg230Cys (C__11720861_10), *APOE* T388C (C__3084793_20) and C526T (C__904973_10) polymorphisms by a real-time polymerase chain reaction (RT-PCR) technique in a 96-well StepOnePlus thermocycler (Applied Biosystems, Life Technologies). Component amounts for each genotyping reaction were 12.5 μL of 2× TaqMan Genotyping Master Mix, (Thermo Fisher Scientific, Waltham, MA, USA), 1.25 μL of 20× Genotyping Assay mix, 3.25 μL of gDNA sample and 8 μL of nuclease-free water for a total volume of 25 μL per well. The thermocycler conditions were 60 °C for 30 s, 95 °C for 10 min, and 40 cycles of denaturation at 92 °C for 15 s and annealing/extension at 60 °C for 1 min. A negative template control (NTC) containing no genomic DNA (gDNA) as well as positive template controls using gDNA samples previously genotyped for each of the three possible combinations of alleles were included in the 96-well plate. Likewise, 10% of the total samples were selected at random for a re-run test, which was 100% concordant with the previous result.

### 2.3. Copy Number Variation

The *AMY1* diploid gene copy number was determined by duplex quantitative RT-PCR using both the TaqMan *AMY1* copy number assay (Hs07226362_cn; Applied Biosystems, Life Technologies) and TaqMan copy number reference assay for human *RNase P* (Applied Biosystems, Life Technologies) in a 96-well StepOnePlus thermocycler (Applied Biosystems, Life Technologies). The components for the duplex reaction were 10 μL of 2× TaqMan Genotyping Master Mix, 1 μL of 20X TaqMan *AMY1* Copy Number Assay, 1 μL of TaqMan copy number reference assay *RNase P*, 6 μL of nuclease-free water and 2 μL of gDNA, for a total volume of 20 μL per well. Four replicates of each gDNA sample were loaded as recommended by the manufacturer for subsequent quantification analyses, and four wells containing NTC were also included in each plate. Universal PCR cycling conditions were configured to run the plates.

StepOne Software v2.1 (Applied Biosystems, Life Technologies) was used for the post-PCR plate read and allelic discrimination analysis of *MTHFR, ABCA1* and *APOE* genotypes. The genotype and allele frequencies were subsequently determined by a simple counting method. The *AMY1* copy number values for each sample were calculated by a relative quantitation algorithm with the Applied Biosystems CopyCaller Software v2.1, (Thermo Fisher Scientific). The median ΔCт of the samples run, as well as an *AMY1* copy number of 6 was configured as calibrator sample settings. The quality metrics of the software (confidence and z-score metrics) were used to validate the assigned copy number.

### 2.4. Geographic Heat Maps

Data was registered in Excel 2010 (Microsoft, Redmond, WA, USA) for further analysis. In separate spreadsheets, all data of the genotype and allele frequencies reported in the previous studies and those determined in the present study were compiled for each polymorphism. Using ArcGIS10 (Ersi, Redlands, CA, USA) software, the geographic distribution of the prevalence of genotypes containing adaptive alleles was represented on a heat map of Mexico, divided by state and geographic region: North (N), Central West (CW), Central East (CE), South (S) and South East (SE) as reported by Moreno-Estrada [21]. The International Terrestrial Reference Frame 1992 and North America Lambert Conformal Conic Projection were used as a coordinate reference system for the georeferencing of ethnic groups and Mestizo populations.

### 2.5. Statistical Analysis

The difference in the prevalence of DRAG polymorphisms among ethnic groups, Mestizos and Mestizos-EUR from CW Mexico were evaluated with a χ^2^-test. An ANOVA test was used to evaluate the difference in the mean value of *AMY1* copies among these sub-populations. Values of *p* < 0.05 were considered significant.

### 2.6. Ethical Guidelines

This cross-sectional study was conducted at the Department of Molecular Biology in Medicine, Civil Hospital of Guadalajara “Fray Antonio Alcalde” in Guadalajara, Jalisco, Mexico. The local Hospital Ethical Committee (Institutional Review Board # C1-01913) by the guidelines of 2013 Declaration of Helsinki approved this study.

## 3. Results

### 3.1. Prevalence of DRAG Polymorphisms in Mexico

In total, data from 33 studies reporting frequencies for DRAG polymorphisms were included (17 for *MTHFR*, 10 for *APOE*, 3 for *ABCA1*, 2 for *LCT*, and 1 for *AMY1*). DRAG polymorphisms have been studied both in Mexican Mestizos and Amerindian populations (ethnic groups), except for *AMY1* copy number, which had only been evaluated in Mestizos. Genotype and allele frequencies of each DRAG polymorphism in ethnic and Mestizo populations (data from the literature review and this study) are listed in Tables S1–S4.

#### 3.1.1. *MTHFR* C677T

**Figure 1:**
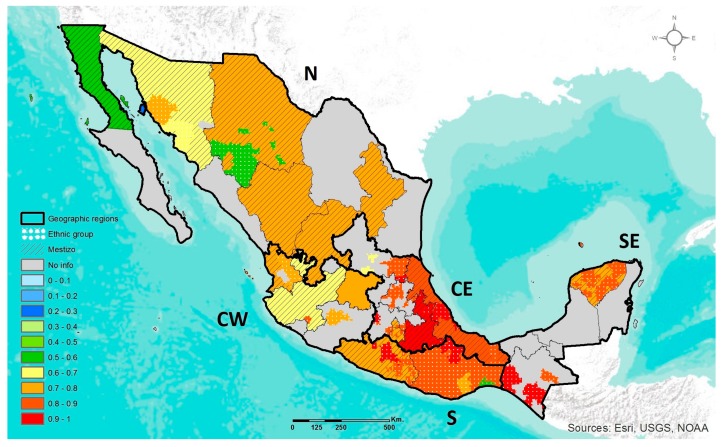
Geographic distribution of the *MTHFR* 677CT+TT genotypes frequency in ethnic groups and Mestizo population of Mexico. N, North; CW, Central West; CE, Central East; S, South; SE, South East.

The most studied DRAG polymorphism in Mexico was the *MTHFR* C677T, of which prevalence information is available for populations in 21 of the country’s 32 states, distributed among the five geographic regions (Table S1). Figure 1 shows the geographic distribution of the frequency of *MTHFR* 677CT+TT genotypes. In general, more than 50% of individuals in both ethnic and Mestizo populations have a genotype containing the T adaptive allele (CT or TT). An exception was the Seri ethnic group in the N region, which had a CT+TT frequency of 26%. Among Mestizos, the lowest prevalence of CT+TT was 58% in a northern population (Baja California), while the highest was 93% in a CE population (Puebla). The prevalence ranges of the CT+TT genotypes observed in Mestizos by geographic region were 58–77% in the N, 59–78% in CW, 76–93% in CE, 81% in the S, and 74–76% in SE. Thus, the frequency range of the T allele in Mestizos was 38–75%. In ethnic groups, not considering the Seris, the lowest prevalence of CT+TT genotypes was 54% in Tarahumaras of the N, and the highest was 100% among Mazahua (CE), Triki (S), Kaqchikel (SE) and Mocho (SE). In general, among ethnic groups, the frequency range of T allele was 32–92%.

In CW Mexico, the ethnic groups evaluated in the present study showed a significantly higher prevalence of *MTHFR* 677CT+TT genotypes compared to Mestizos and Mestizos-EUR (Table 2).

#### 3.1.2. *ABCA1* R230C

**Figure 2:**
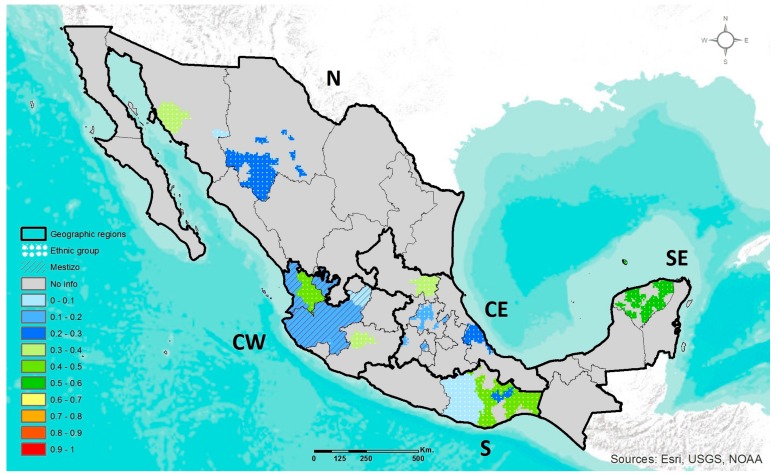
Geographic distribution of the *ABCA1* 230RC+CC genotypes frequency in ethnic groups and Mestizo population of Mexico.

The *ABCA1* R230C polymorphism has been studied in populations of 12 states distributed among the five geographic regions, but mostly in AM ethnic groups (Table S2). The geographic distribution of the frequency of *ABCA1* 230RC+CC genotypes is depicted in Figure 2. Prevalence of the genotypes in Mestizos was only previously reported from CE region, and thus herein the prevalence in Mestizos from CW was added. Among the Mestizos of these two geographic regions, the prevalence range of genotypes containing the C adaptive allele (RC+CC) was 6–15% in CW and 20% in CE, while the frequency range of C allele was 3–8% and 11% respectively. In ethnic groups, the highest prevalence of the RC+CC genotypes was 55% in Mayas (SE), while the lowest was 17% in Otomis (CE). However, the Pima and Seri groups in N showed a very different prevalence (8% and 0%) compared to other ethnic groups. Also, the prevalence of 0% reported for Mixtecos (S) was not considered in further analyses, since the sample number of subjects was only four. Thus, the frequency range of C allele among ethnic groups was 8–29%, not considering the groups noted above.

Among the sub-populations of CW Mexico evaluated in this study, the AM ethnic groups showed a significantly higher prevalence of *ABCA1* 230RC+CC genotypes compared to Mestizos and Mestizos-EUR (Table 2).

#### 3.1.3. *APOE* ε2, ε3, ε4

**Figure 3:**
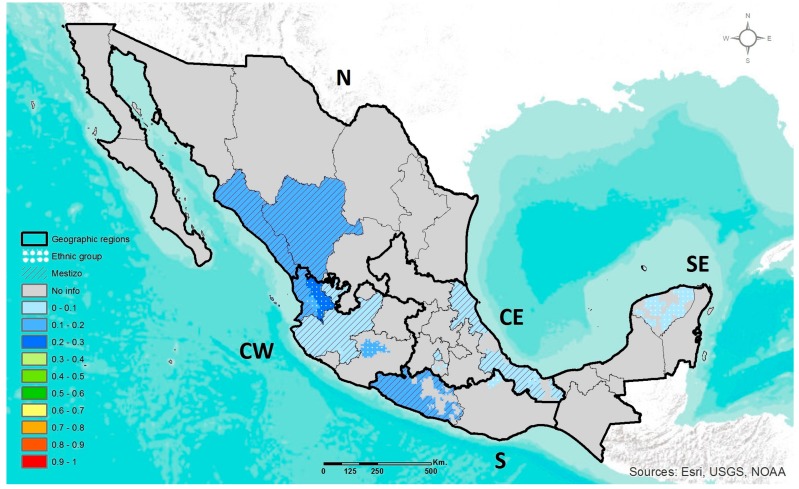
Geographic distribution of *APOE* ε4 allele in ethnic groups and Mestizo population of Mexico.

The frequencies of *APOE* polymorphisms have been studied in 11 States of the country, also distributed among the five geographic regions (Table S3). The *APOE* ε3 allele was the most frequent in Mestizos and AM ethnic groups, with a frequency range of 76–96% and 71–94%, respectively. The *APOE* ε2 allele was nearly absent in AM ethnic groups, where 3% was the highest frequency observed of this allele in Nahuas (CW). However, among Mestizos, the highest frequency of the ε2 allele was 9% in a population of Mestizos-EUR (CW) evaluated in this study. Only the frequency of the ε4 adaptive allele is displayed in Figure 3. The highest frequency of the ε4 allele in Mestizos was 18% in the SE region, while the lowest was 3% in two populations of Mestizos-EUR (CW) evaluated in this study. The prevalence ranges of the ε4 allele by geographic region were 12–16% in N, 3–11% in CW, 8–10% in CE, and 18% in SE; it has not been evaluated in Mestizos of S region. Among ethnic groups, Huicholes (CW) presented the highest frequency of the ε4 allele (29%), whereas Nahuas (CE) showed the lowest frequency (5%).

Considering only the populations evaluated in this study, in CW Mexico the ethnic groups had a significantly higher prevalence of the ε4 allele compared to Mestizos and Mestizos-EUR (Table 2).

#### 3.1.4. *LCT* C-13910T

**Figure 4:**
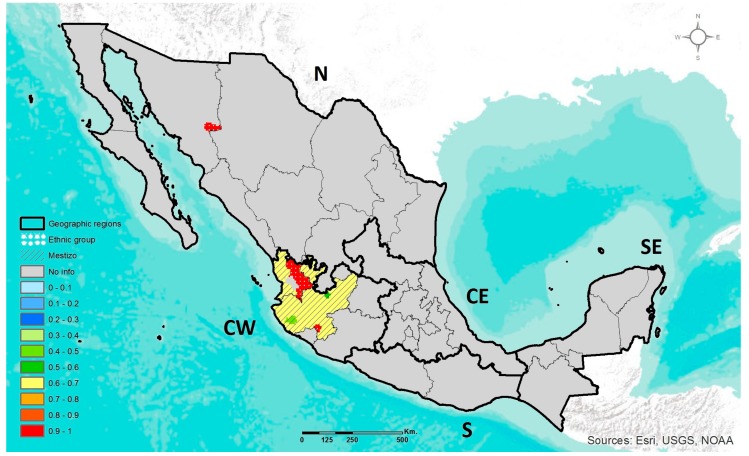
Geographic distribution of the *LCT*-13910CC genotype frequency in ethnic groups and Mestizo population of Mexico.

The *LCT* C-13910T polymorphism has only been evaluated in the CW Mexico region, in a previous study by our research group. However, another study was found that reports the prevalence of this polymorphism in the Pima (N) ethnic group (Table S4). In CW Mestizos, the prevalence range of genotypes containing the T adaptive allele related to lactase persistence (*LCT*-13910 CT+TT) was 31–56%, and the frequency range of T allele was 16–33%. A subpopulation of Mestizos-EUR was the one with the highest prevalence of CT+TT genotypes (56%). Nonetheless, the T allele was rare among AM ethnic groups. Only the Nahuas presented an allele frequency of 1%. The geographic distribution of the *LCT*-13910CC genotype related to lactase non-persistence (LNP) is illustrated in Figure 4. A heterogeneous distribution of LNP-related CC genotype was observed in the CW region. The highest prevalence of the CC genotype (98–100%) was observed among AM ethnic groups, while the lowest was among the Mestizos-EUR subgroup (44–55%).

#### 3.1.5. *AMY1* Copy Number Variation

**Figure 5:**
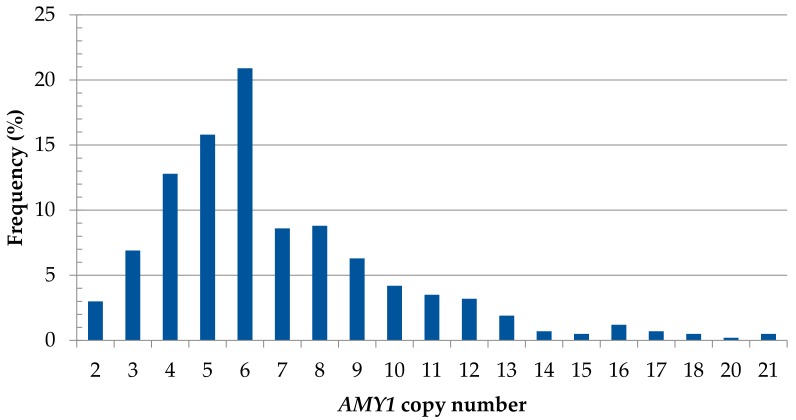
Distribution of the *AMY1* gene diploid copy number in general adult population of CW Mexico.

In the single study found of the *AMY1* gene, the copy number variation was evaluated in children with obesity and normal weight randomly selected from five different Mexican States. The mean number of *AMY1* copies was 6.1 ± 1.9 in children with obesity and 7.0 ± 2.7 in those with normal weight, with a general range of 1 to 16 copies [26]. In the present study, the *AMY1* diploid copy number was evaluated for the first time in different adult subpopulations of CW Mexico. The mean *AMY1* diploid copy number of these subpopulations is shown in Table 2. There was no significant difference in the mean number of *AMY1* copies among the ethnic groups, Mestizos, and Mestizos-EUR. In general, in the population of CW (*n* = 431), *AMY1* ranged from 2 to 21 copies, with a mean diploid number of 6.82 ± 3.3, and 61.5% of the individuals presented ≥6 copies in their genome (Figure 5).

## 4. Discussion

It has been documented that populations abandoning their traditional diets are prone to health problems. Nowadays, diet-related genetic adaptations fixed over the course of gene-culture co-evolutionary processes have been associated with the risk of chronic diseases [27,28]. Therefore, the DRAG polymorphisms analyzed herein were selected because previous studies have reported them with signatures of positive selection associated with ancestral dietary practices, as summarized in Table 1 [10,12,18,19,20]. Also, association studies have shown that in the current Westernized diet environment, these polymorphisms are associated with disease risk when carriers are exposed to a different nutritional environment.

In the present study, all suitable data published to date about the prevalence of some DRAG polymorphisms in Mexican population was compiled. This allowed for an overview of the frequency of *MTHFR* (rs1801133), *ABCA1* (rs9282541) and *APOE* (rs429358/rs7412) polymorphisms in Mestizos and ethnic groups across the country. However, so far *LCT* (rs4988235) and *AMY1* polymorphisms have only been studied in adults of CW Mexico. In general, the N region of the country had the least data regarding the prevalence of DRAG polymorphisms.

Throughout Mesoamerica in the central and southern regions of ancient Mexico, the main pre-Hispanic cultures consumed a wide variety of endemic wild plants and later their domesticated counterparts which became the staple foods of the pre-Hispanic diet [29]. The development of “milpa” agriculture containing mixed crops with complementary nutritional values, gave rise to a new food-culture environment that could have influenced the selection of DRAGs [15]. In this study, the highest prevalence of the *MTHFR* 677T, *ABCA1* 230C, and *APOE* ε4 adaptive alleles were observed among ethnic groups located in the central (CE and CW) and south (SE and S) regions of Mexico. Nevertheless, Mestizos also presented a high frequency of the adaptive alleles. These results are in line with studies analyzing the present-day genetic structure pattern of Mexicans, which state that despite the genetic admixture that occurred after the European Conquest, the ancestral AM component remains representative. In Mestizos, this AM ancestry shows a gradient increasing from north-to-south, notably higher in the ethnic groups situated in the central and south regions of Mexico [21]. Thus, the variable proportion of the AM ancestry could be contributing to the high or heterogeneous frequency of the DRAG polymorphisms observed in AM ethnic groups and Mestizos, respectively.

It has been proposed that the selection of the *MTHFR* 677T allele could be related to environmental factors such as high availability of dietary folate that allowed for normal metabolism, despite the encoded thermolabile enzyme with reduced enzymatic activity [30,31]. In the Mexican population, the selection of the T allele might have been influenced by the pre-Hispanic diet through the frequent consumption of the leafy green vegetables known as “quelites”, and other folate-rich foods such as avocado, amaranth and kidney beans. In this study, it was observed that more than 50% of both Mexican Mestizo and ethnic populations are T allele carriers (CT or TT). Nonetheless, the wide range of the *MTHFR* 677T allele frequency which was lower in the northern ethnic groups (32%) and higher in the South (92%) (Table S1) may be explained by the fact that the northern ethnic groups have long lived in the desert climate of Aridoamerica, a region with quite different ecological features compared to Mesoamerica in which the consumption of leafy greens would be more limited, thus affecting the frequency of the adaptive allele. However, in the current nutritional environment, with a low intake of folate-rich foods, being a T allele carrier confers a higher risk for health-related conditions, such as cardiovascular disease, certain cancers and nonalcoholic steatohepatitis [32,33,34,35,36]. Furthermore, it has been suggested that the folate recommended dietary allowance (RDA) of 400 μg/day as dietary folate equivalents (DFE) for adult women and men may be inadequate for T allele carriers [37]. It has been recommended that the RDA be increased up to 575–830 μg DFE/day for T allele carriers, the majority of the Mexican population [38,39].

*ABCA1* (rs9282541) and *APOE* (rs429358/rs7412) polymorphisms are involved in lipid metabolism and are important determinants of plasma lipid levels. The *ABCA1* 230C allele is related to low plasma levels of high-density lipoprotein cholesterol (HDL-C), while *APOE* ε4 is associated with elevated levels of total cholesterol and low-density lipoprotein cholesterol (LDL-C) [40,41]. Hence, it has been suggested that the selection of both alleles might have favored a metabolism tending toward energy storage [42,43]. Estimates of the age of the *ABCA1* 230C allele are around 7540 years before present, compatible with an American continental origin. It has been documented that the 230C allele might have been important for survival throughout the colonization of the continent, as well as for the Mesoamerican village lifestyle during early maize domestication, where plantation losses might have been common. In line with this hypothesis is the fact that among the Mexican population, the highest prevalence of the 230C and ε4 alleles were found in native Huicholes and Coras (CW), descendants of nomadic ethnic groups and who live a physically active lifestyle [19]. In contrast, the APOE ε2 allele was found mainly in the groups with elevated EUR ancestry, also in agreement with its EUR-related origin.

Moreover, the pre-Hispanic diet was also scarce in animal meat with high cholesterol and saturated fat content. Furthermore, in Mesoamerica, there was no pork, chicken, cattle and derived foods such as dairy. This last feature is consistent with the extremely low prevalence of the lactase persistence *LCT*-13910T allele in AM groups, while on the other hand its highest prevalence was observed in Mestizos-EUR from CW with high EUR ancestry. However, nowadays, Mexicans have an hepatopathogenic dietary pattern characterized by high intake of total fatty acids (>30%), saturated fatty acids (>7%), dietary cholesterol (>200 mg/day), simple carbohydrates (>10%) and an imbalanced n-6:n-3 polyunsaturated fatty acids (PUFA) ratio (12:1). It is also deficient in nutrients with antioxidant properties such as vitamins A (<900 μg/day) and E (<10 mg/day), magnesium (<350 mg/day) and zinc (<15 mg/day). Overall, this dietary pattern has been associated with abnormal intrahepatic lipid accumulation and the onset and progression of liver damage [44,45,46,47]. In this modern environment, the *ABCA1*, *APOE* (ε2 and ε4) and *LCT* polymorphisms have been associated with obesity, dyslipidemias, type 2 diabetes and other metabolic alterations [24,48,49,50,51,52,53]. However, it was demonstrated that the polymorphism-disease association is not replicated when studying Native American populations that follow their traditional diet and lifestyle [54]. Thus, the genetic susceptibility to the development of these abnormalities can be modulated by environmental factors. For example, clinical trials in *APOE* ε4 or *ABCA1* 230C allele carriers following the most stringent dietary recommendations (<30% of energy from fat (7% saturated fatty acids), 15–18% from protein and 50–55% from total carbohydrates, as well as no more than 240 mg/day of dietary cholesterol) have shown an effective response on serum lipids [55,56,57].

The *AMY1* gene copy number was evaluated for the first time in Mexican adult population from CW region. In general, more than 60% of the population presented an *AMY1* diploid copy number of ≥6 with a mean of 6.82 ± 3.3 copies. When *AMY1* copies were analyzed by subpopulation, no difference in the mean of *AMY1* copies was observed among ethnic groups, Mestizos, and Mestizos-EUR. This data is consistent with the *AMY1* copy numbers in agricultural societies reported by Perry et al., who reported that agricultural societies traditionally consuming a high-starch diet showed a higher mean of *AMY1* diploid copies than pastoralist/fishing societies with low-starch diet (6.72 ± 2.35 vs. 5.44 ± 2.04) [12]. Therefore, we can infer that the average of *AMY1* copies in Mexican population is in accordance with their history as an agricultural society, with low-glycemic index starchy foods such as maize and derivatives, kidney beans, amaranth among others, comprising a considerable proportion of their pre-Hispanic diet as the source of carbohydrates, contrary to the modern-day dietary pattern containing more simple carbohydrates. Currently, studies that have evaluated the relation among *AMY1* copies and obesity have obtained variable results according to their methodology. While some have reported that a reduced *AMY1* diploid copy number (<8) is associated with an increased body mass index (BMI) and risk of metabolic abnormalities, a recent study that highlights its higher-resolution analysis (molecular and computational) reported no association among *AMY1* and BMI or obesity. Thus, the relation among *AMY1* copies and BMI remains controversial and has been explained by differences among populations, the complex context of the genetic-environmental interplay leading them to obesity and the study designs [26,58,59,60].

The subpopulations of CW Mexico selected in this study are representative of the overall genetic heterogeneity of the Mexicans. The prevalence of the adaptive alleles of *MTHFR*, *ABCA1*, and *APOE* was significantly higher in the ethnic groups compared with Mestizos with intermediate AM ancestry and Mestizos-EUR, as expected (Table 2). In addition, this distribution was in agreement with the population stratification due to historical events and economic inequality as shown in previous studies [20,61]. In this region, Mestizos with a strong EUR ancestry and cultural heritage congregate in the metropolitan areas and towns, in which the ancestral AM culture has a weaker impact on modern-day practices. In contrast, the rural Mestizos with higher AM lineage and ethnic groups, which are geographically dispersed, may still follow traditional lifestyles to a larger extent [15].

The fight against morbidity and mortality due to NR-NCD has been tackled in some countries using traditional diets or regional foods as in the case of the Mediterranean-type diet [62,63,64]. However, each ecoregion contains its own biodiversity with differences in genotypic frequencies, food products, and food culture. At this point, it is noteworthy to highlight that given the similar prevalence profile of the AM adaptive alleles among the studied Mestizo populations, the current hepatopathogenic dietary pattern is discordant with the AM genetic background. Hence, to revert this unhealthy diet, a preliminary proposal would be to follow a pre-Hispanic diet to aid the folate deficiency seen in many Mexicans, lower the ingestion of saturated fat/cholesterol-rich foods, avoid dairy, and consume low-glycemic index starchy foods as a source of carbohydrates.

An integrative approach between nutrigenetic, agronomy/culinary and behavioral nutrition research is required to provide the knowledge that we need to prevent and treat NR-NCD [65,66,67]. Therefore, the prevalence profile of the DRAG polymorphisms presented herein as well as others previously assessed may aid to define the genetic diversity of the Mexican genome and its linkage to NR-NCD [68,69,70,71]. Alongside this, the pre-Hispanic food culture, given an award in 2010 as an Immaterial Patrimony of Humanity is a valuable resource, not only due to its nutritional properties but also because it holds the legacy of the forefathers who practiced more eco-friendly agriculture and transmitted the family traditions and rituals to new generations as well [72]. The challenges that Mexico and other Latin American countries are facing is precisely the loss of biodiversity due to overexploitation of the monoculture farming and food acculturation.

## 5. Conclusions

The highest prevalence of the adaptive alleles of the DRAG polymorphisms was observed in the ethnic groups followed by the Mestizos with an intermediate AM ancestry. This suggests that being carriers of the adaptive alleles may require the nutritional needs related to the dietary practices in which they were adapted in order to avoid the risk of disease to which currently they have been associated. Given these genetic differences, genome-based nutritional advice should be tailored in a regionalized and individualized manner according to the available foods and the pre-Hispanic traditional food culture that may lead to a healthier dietary pattern as mentioned above. More so, strategies based on the knowledge of the gene-culture co-evolutionary processes, prevalence profile, and the implementation of associational and interventional studies are warranted.

Finally, the data compiled in this study may be useful for identifying the subpopulations that may be at an increased risk for the development of NR-NCD in the current nutrition transition, and likewise to understand why some areas of the country show a high prevalence of these diseases. However, further studies to increase the data regarding the prevalence of the DRAG polymorphisms from other regions of the country are required.

## Figures and Tables

**Table 1:** Diet-related adaptive gene (DRAG) polymorphisms evaluated in this study.

Gene	Function of the Encoded Protein	Polymorphism	Adaptive Allele	Cultural Selection Pressure	Biological Outcome	Population
*MTHFR*	Folate and homocysteine metabolism	C677T (rs1801133)	T	High intake of folate-rich foods such as leafy greens	A normal metabolism despite a thermolabile enzyme	Amerindians
*ABCA1*	Interacts with ApoA1 to form nascent HDL	Arg230Cys (rs9282541)	Cys	Maize domestication, low-fat diet	Cholesterol efflux reduction, energy storage	Amerindians
*APOE*	Structural and metabolism-regulatory function of lipoproteins	T388C (rs429358) C526T (rs7412) ε2, ε3, ε4	ε4	Low cholesterol and low saturated fat diet	Greater affinity to its receptors, energy storage	Amerindians Sub-Saharans
*LCT*	Lactose digestion	C-13910T (rs4988235)	T	Pastoralism, consumption of milk and derivates	Adult lactase persistence	Europeans
*AMY1*	Starch hydrolysis	Copy number variation	≥6 copies	Farming, rich-starch diets	Higher enzyme expression, better starch digestion	Europeans Japanese Hazda hunter-gatherers

***MTHFR***: methylenetetrahydrofolate reductase; ***ABCA1***: ATP-binding cassette transporter A1; **ApoA1**: apolipoprotein A1; **HDL**: high-density lipoprotein; ***APOE***: apolipoprotein E; ***LCT***: lactase gene; ***AMY1***: salivary amylase 1 gene.

**Table 2:** Genotype frequency of DRAG polymorphisms in subpopulations of Central West Mexico

	Genotypes/Alleles	Population			
		Ethnic Groups n (%)	Mestizos n (%)	Mestizos-EUR n (%)	*p*
*MTHFR* C677T	Number	179 ^a^	952 ^b^	163 ^c^	
	CC	37 (20.7)	280 (29.4)	57 (35.0)	
	CT+TT	142 (79.3)	672 (70.6)	106 (65.0)	0.022 *
*ABCA1* R230C	Number	176 ^a^	540 ^b^	194 ^d^	
	RR	118 (67.0)	463 (85.7)	170 (87.6)	
	RC+CC	58 (33.0)	77 (14.3)	24 (12.4)	7.0 × 10^−7^ *
*APOE* ε2, ε3, ε4	Number	182 ^a^	637 ^b^	64 ^e^	
	ε2 (2n)	0 (0.0)	59 (4.6)	7 (5.5)	
	ε3 (2n)	290 (79.7)	1094 (85.9)	117 (91.4)	
	ε4 (2n)	74 (20.3)	121 (9.5)	4 (3.1)	3.0 × 10^−8^ *
*AMY1*	Number	137 ^a^	146 ^b^	148 ^d^	
	Copy number	6.77 ± 3.5	6.99 ± 3.3	6.71 ± 3.1	
	(Χ ± SD)				0.794 **

^a^ Ethnic groups (Nahua + Huichol); ^b^ Mestizos with intermediate AM ancestry (Tepic, Nayarit + Guadalajara, Jalisco); ^c^ Mestizos-EUR (Cuquio, Jalisco + Villa Purificación, Jalisco); ^d^ Mestizos-EUR (Cuquio, Jalisco + Villa Purificación, Jalisco + San Miguel el Alto, Jalisco); ^e^ Mestizos-EUR (Villa Purificación, Jalisco + San Miguel el Alto, Jalisco); * χ^2^-test, ethnic groups vs. Mestizos and Mestizos-EUR; ** ANOVA.

## Supplementary Materials

The following are available online at www.mdpi.com/2075-4426/7/4/16/s1, Table S1: Genotype and allele frequencies of the *MTHFR* C677T polymorphism (rs1801133) in ethnic groups and Mestizo population of Mexico. Table S2: Genotype and allele frequencies of the *ABCA1* Arg230Cys (rs9282541) polymorphism in ethnic groups and Mestizo population of Mexico. Table S3: *APOE* allele frequencies in ethnic groups and Mestizo population of Mexico. Table S4: Genotype and allele frequencies of the *LCT* (*MCM6*) C-13910T (rs4988235) polymorphism in ethnic groups and Mestizo population of Mexico. References [73,74,75,76,77,78,79,80,81,82,83,84,85,86,87,88,89,90,91,92] are cited in the supplementary materials.
