# Supplementary material for: Tailoring Nutritional Advice for Mexicans Based on Prevalence Profiles of Diet-Related Adaptive Gene Polymorphisms
Source: J Pers Med. 2017 Nov 10;7(4):16. doi: 10.3390/jpm7040016 (PMC5748628; doi:10.3390/jpm7040016)
Supplement: Supplementary file 1 [file jpm-07-00016-s001.pdf]

Journal of Personalized Medicine

MDPI

Table S1. Genotype and allele frequencies of the *MTHFR* C677T polymorphism (rs1801133) in ethnic groups and Mestizo population of Mexico

Geographical region	State	Population	N	*MTHFR* C677T genotype (%)			T allele frequency (%)	Reference
				CC	CT	TT		
**North**	Baja California Norte	Mestizo	150	0.42	0.40	0.18	0.38	[73]
	Sonora	Mestizo	166	0.34	0.48	0.17	0.42	[74]
	Sonora	Mayo	28	0.36	0.46	0.18	0.41	[75]
	Sonora	Seri	19	0.74	0.26	0.00	0.13	[75]
	Sonora	Yaqui	37	0.24	0.57	0.19	0.47	[75]
	Sonora	Yaqui	27	0.33	0.41	0.26	0.46	[76]
	Chihuahua	Mestizo	197	0.27	0.61	0.12	0.42	[77]
	Chihuahua	Tarahumara	88	0.46	0.44	0.10	0.32	[75]
	Chihuahua	Tarahumara	38	0.45	0.39	0.16	0.36	[78]
	Durango	Mestizo (women)	194	0.28	0.50	0.22	0.47	[33]
	Zacatecas	Mestizo	181	0.28	0.49	0.23	0.47	[74]
	Nuevo León	Mestizo (women)	41	0.29	0.46	0.25	0.48	{79]
	Nuevo León	Mestizo	533	0.26	0.54	0.20	0.47	[80]
	Nuevo León	Mestizo (men)	60	0.23	0.62	0.15	0.46	[81]
**Central West**	Nayarit	Mestizo	184	0.28	0.50	0.22	0.47	*
	Nayarit	Huichol	95	0.25	0.43	0.32	0.53	*
	Nayarit	Huichol	15	0.20	0.53	0.27	0.53	[76]
	Nayarit	Huichol	50	0.16	0.56	0.28	0.56	[78]
	Jalisco	Mestizo	101	0.31	0.50	0.19	0.44	[78]
	Jalisco	Mestizo	170	0.35	0.46	0.19	0.42	[34]
	Jalisco	Mestizo (Gdl)	768	0.30	0.51	0.20	0.45	*
	Jalisco	Mestizo-EUR (Cuq)	131	0.34	0.44	0.23	0.45	*
	Jalisco	Mestizo-EUR (V.P.)	32	0.41	0.40	0.19	0.39	*
	Jalisco	Nahua	84	0.16	0.44	0.41	0.63	*

	Guanajuato	Mestizo	**184**	**0.22**	**0.53**	**0.24**	**0.51**	**[74]**
	Michoacán	Purépecha	**29**	**0.07**	**0.45**	**0.48**	**0.71**	**[76]**
	Michoacán	Purépecha	**21**	**0.19**	**0.48**	**0.33**	**0.57**	**[78]**
	Michoacán	Purépecha	**14**	**0.43**	**0.43**	**0.14**	**0.36**	**[75]**
**Central East**	San Luis Potosí	Nahua	**44**	**0.09**	**0.52**	**0.39**	**0.65**	**[75]**
	San Luis Potosí	Huasteco	**79**	**0.11**	**0.41**	**0.48**	**0.68**	**[75]**
	San Luis Potosí	Pame	**10**	**0.30**	**0.70**	**0.00**	**0.35**	**[75]**
	Hidalgo	Otomí	**220**	**0.13**	**0.38**	**0.49**	**0.68**	**[75]**
	Mexico State	Mazahua	**10**	**0.00**	**0.60**	**0.40**	**0.72**	**[75]**
	Mexico State	Nahua	**22**	**0.14**	**0.45**	**0.41**	**0.64**	**[75]**
	Mexico City	Mestizo	**71**	**0.24**	**0.46**	**0.30**	**0.53**	**[76]**
	Mexico City	Nahua	**150**	**0.07**	**0.35**	**0.59**	**0.76**	**[74]**
	Mexico City	Nahua	**52**	**0.13**	**0.48**	**0.38**	**0.63**	**[75]**
	Morelos	Mestizo (women)	**130**	**0.22**	**0.52**	**0.26**	**0.52**	**[82]**
	Morelos	Mestizo (women)	**74**	**0.21**	**0.53**	**0.26**	**0.52**	**[83]**
	Morelos	Nahua	**44**	**0.14**	**0.59**	**0.27**	**0.57**	**[75]**
	Puebla	Mestizo	**28**	**0.07**	**0.36**	**0.57**	**0.75**	**[84]**
	Puebla	Nahua	**52**	**0.06**	**0.48**	**0.46**	**0.70**	**[75]**
	Veracruz	Mestizo	**182**	**0.18**	**0.52**	**0.30**	**0.56**	**[74]**
	Veracruz	Totonaco	**97**	**0.08**	**0.35**	**0.57**	**0.74**	**[75]**
	Veracruz	Totonaco	**25**	**0.08**	**0.56**	**0.36**	**0.64**	**[74]**
	Veracruz	Popoluca de la Sierra	**36**	**0.14**	**0.50**	**0.36**	**0.61**	**[75]**
**South**	Guerrero	Mestizo	**181**	**0.19**	**0.48**	**0.33**	**0.57**	**[74]**
	Guerrero	Mestizo (women)	**112**	**0.22**	**0.51**	**0.27**	**0.52**	**[85]**
	Guerrero	Mestizo (women)	**196**	**0.17**	**0.44**	**0.39**	**0.61**	**[86]**
	Guerrero	Mixteco (women)	**124**	**0.10**	**0.40**	**0.50**	**0.70**	**[86]**
	Guerrero	Nahua (women)	**135**	**0.05**	**0.43**	**0.52**	**0.73**	**[86]**
	Oaxaca	Chinanteco	**81**	**0.05**	**0.27**	**0.68**	**0.81**	**[75]**
	Oaxaca	Mazateco	**59**	**0.07**	**0.36**	**0.58**	**0.75**	**[75]**
	Oaxaca	Mixteco	**134**	**0.11**	**0.30**	**0.59**	**0.74**	**[75]**

	Oaxaca	Mixe	**89**	**0.13**	**0.48**	**0.38**	**0.62**	**[75]**
	Oaxaca	Zapoteco	**66**	**0.06**	**0.29**	**0.65**	**0.80**	**[75]**
	Oaxaca	Zapoteco	**42**	**0.17**	**0.21**	**0.62**	**0.73**	**[74]**
	Oaxaca	Chontal de Oaxaca	**44**	**0.23**	**0.36**	**0.41**	**0.59**	**[75]**
	Oaxaca	Huave	**26**	**0.42**	**0.50**	**0.08**	**0.33**	**[75]**
	Oaxaca	Trikis	**89**	**0.00**	**0.16**	**0.84**	**0.92**	**[76]**
**South East**	Chiapas	Kaqchikel	**36**	**0.00**	**0.31**	**0.69**	**0.85**	**[75]**
	Chiapas	Mocho	**15**	**0.00**	**0.20**	**0.80**	**0.90**	**[75]**
	Chiapas	Chuj	**17**	**0.06**	**0.24**	**0.71**	**0.82**	**[75]**
	Chiapas	Kanjobal	**29**	**0.07**	**0.31**	**0.62**	**0.78**	**[75]**
	Chiapas	Jakalteco	**40**	**0.08**	**0.25**	**0.68**	**0.80**	**[75]**
	Chiapas	Mam	**45**	**0.09**	**0.38**	**0.53**	**0.72**	**[75]**
	Chiapas	Tojolabal	**46**	**0.13**	**0.61**	**0.26**	**0.57**	**[75]**
	Yucatán	Mestizo	**174**	**0.26**	**0.55**	**0.19**	**0.46**	**[74]**
	Yucatán	Mestizo	**110**	**0.22**	**0.48**	**0.30**	**0.54**	**[87]**
	Yucatán	Maya	**234**	**0.21**	**0.48**	**0.31**	**0.55**	**[75]**
	Yucatán	Maya	**54**	**0.13**	**0.52**	**0.35**	**0.61**	**[76]**

* Genotyped in the present study.

Table S2. Genotype and allele frequencies of the *ABCA1* Arg230Cys (rs9282541) polymorphism in ethnic groups and Mestizo population of Mexico

Geographical region	State	Population	N	*ABCA1* R230C genotype (%)			C allele frequency (%)	Reference
				RR	RC	CC		
**North**	Sonora	Yaqui	45	0.67	0.24	0.09	0.21	[42]
	Sonora	Yaqui	37	0.70	0.19	0.11	0.20	[40]
	Sonora	Pima	2563	0.92	0.07	0.01	0.04	[42]
	Sonora	Seri	87	1.00	0.00	0.00	0.00	[42]
	Chihuahua	Tarahumara	109	0.74	0.21	0.05	0.15	[42]
**Central West**	Nayarit	Mestizo	183	0.87	0.12	0.02	0.07	*
	Nayarit	Huichol	92	0.53	0.40	0.07	0.27	*
	Nayarit	Cora	123	0.50	0.42	0.08	0.29	[42]
	Jalisco	Mestizo (Gdl)	357	0.85	0.15	0.00	0.08	*
	Jalisco	Mestizo-EUR (V.P.)	32	0.88	0.09	0.03	0.08	*
	Jalisco	Mestizo-EUR (Cuq)	129	0.86	0.14	0.00	0.07	*
	Jalisco	Mestizo-EUR (S.M.)	33	0.94	0.06	0.00	0.03	*
	Jalisco	Nahua	84	0.82	0.17	0.01	0.09	*
	Michoacán	Purépecha	35	0.63	0.31	0.06	0.21	[40]
**Central East**	San Luis Potosí	Teneek	67	0.67	0.30	0.03	0.18	[40]
	Hidalgo	Otomí	42	0.83	0.17	0.00	0.08	[42]
	Mexico State	Mazahua	88	0.82	0.17	0.01	0.10	[40]
	Mexico City	Mestizo	429	0.80	0.18	0.02	0.11	[40]
	Veracruz	Nahua	267	0.69	0.28	0.03	0.17	[42]
	Veracruz	Totonaca	113	0.76	0.21	0.03	0.13	[42]
**South**	Oaxaca	Zapoteca	106	0.59	0.40	0.01	0.21	[42]
	Oaxaca	Mixe	19	0.79	0.21	0.00	0.11	[19]
	Oaxaca	Mixteco	4	1.00	0.00	0.00	0.00	[19]
**South East**	Yucatán	Maya	40	0.45	0.52	0.03	0.28	[40]

* Genotyped in the present study.

Table S3. *APOE* allele frequencies in ethnic groups and Mestizo population of Mexico

Geographical region	State	Population	N	*APOE* alleles (%)			Reference
				E2	E3	E4	
**North**	Durango	Mestizo	**30**	**0.00**	**0.88**	**0.12**	**[20]**
	Sinaloa	Mestizo	**101**	**0.06**	**0.78**	**0.16**	**[88]**
**Central West**	Nayarit	Mestizo	**61**	**0.02**	**0.87**	**0.11**	**[20]**
	Nayarit	Mestizo	**110**	**0.03**	**0.87**	**0.10**	*
	Nayarit	Huichol	**40**	**0.00**	**0.71**	**0.29**	**[20]**
	Nayarit	Huichol	**99**	**0.00**	**0.73**	**0.27**	*
	Nayarit	Huichol	**31**	**0.00**	**0.76**	**0.24**	**[89]**
	Nayarit	Cora	**44**	**0.00**	**0.84**	**0.16**	**[89]**
	Jalisco	Mestizo	**179**	**0.08**	**0.84**	**0.08**	**[20]**
	Jalisco	Mestizo	**370**	**0.08**	**0.84**	**0.08**	**[51]**
	Jalisco	Mestizo (Gdl)	**527**	**0.05**	**0.86**	**0.09**	*
	Jalisco	Mestizo-EUR (V.P.)	**32**	**0.01**	**0.96**	**0.03**	*
	Jalisco	Mestizo-EUR (S.M.)	**32**	**0.09**	**0.88**	**0.03**	*
	Jalisco	Nahua	**83**	**0.03**	**0.87**	**0.10**	*
	Michoacán	Purépecha	**142**	**0.02**	**0.80**	**0.18**	**[54]**
**Central East**	Mexico City	Mestizo	**224**	**0.03**	**0.87**	**0.10**	**[89]**
	Mexico City	Mestizo	**278**	**0.03**	**0.89**	**0.08**	**[41]**
	Mexico City	Mestizo	**83**	**0.00**	**0.92**	**0.08**	**[90]**
	Morelos	Nahua	**186**	**0.01**	**0.90**	**0.09**	**[91]**
	Veracruz	Mestizo	**80**	**0.00**	**0.91**	**0.09**	**[89]**
	Veracruz	Nahua	**120**	**0.01**	**0.94**	**0.05**	**[89]**
**South**	Guerrero	Mestizo (women)	**400**	**0.06**	**0.76**	**0.18**	**[50]**
	Oaxaca	Mazateco	**75**	**0.00**	**0.90**	**0.10**	**[90]**
**South East**	Yucatán	Maya	**135**	**0.00**	**0.91**	**0.09**	**[92]**

* Genotyped in the present study.

Table S4. Genotype and allele frequencies of the *LCT* (*MCM6*) C-13910T (rs4988235) polymorphism in ethnic groups and Mestizo population of Mexico

Geographical region	State	Population	N	*LCT (MCM6)* C-13910T genotype (%)			T allele frequency (%)	Reference
				CC	CT	TT		
**North**	Sonora	Pima	**50**	**1.00**	**0.00**	**0.00**	**0.00**	**[10]**
**Central West**	Nayarit	Mestizo	**185**	**0.69**	**0.31**	**0.00**	**0.16**	**[24]**
	Nayarit	Huichol	**95**	**1.00**	**0.00**	**0.00**	**0.00**	**[24]**
	Jalisco	Mestizo-EUR (V.P.)	**32**	**0.44**	**0.47**	**0.09**	**0.33**	**[24]**
	Jalisco	Mestizo-EUR (Cuq)	**99**	**0.55**	**0.37**	**0.08**	**0.27**	**[24]**
	Jalisco	Mestizo (Gdl)	**699**	**0.64**	**0.32**	**0.04**	**0.20**	**[24]**
	Jalisco	Nahua	**86**	**0.98**	**0.02**	**0.00**	**0.01**	**[24]**
